# Interferon-γ and interleukin-17A associations with vascular dysfunction following paediatric cardiac surgery with cardiopulmonary bypass

**DOI:** 10.1017/S1047951126111779

**Published:** 2026-03-27

**Authors:** Matthew Aaron Solomon, Milene Fontes, Luke Krispinsky, James C. Slaughter, Chloe Barreto-Massad, Fred S. Lamb, Ryan Stark

**Affiliations:** 1 Pediatrics, Vanderbilt University Medical Center, USA; 2 Biostatistics, Vanderbilt University Medical Center, USA; 3 Vanderbilt University, USA

**Keywords:** Cardiopulmonary bypass, cardiac surgical procedures, endothelial cells, CHD

## Abstract

**Background:**

Cardiopulmonary bypass-induced inflammation is associated with poor postoperative outcomes. Bypass exposure has been associated with shifts in lymphocyte populations. This study aimed to describe two cytokine profiles associated with T and NK cells and their effects on clinical markers of postoperative cardiovascular dysfunction in children undergoing cardiac surgery with cardiopulmonary bypass.

**Methods:**

Children from two major children’s hospitals undergoing corrective cardiac surgery with cardiopulmonary bypass were included. Plasma was collected pre-, 0 to 4 hours post- and 24 hours (when available) postoperatively. Plasma concentrations of cytokines were quantified using enzyme-linked immunosorbent assays. Delta cytokine concentrations were compared to vasoactive infusion score and percent fluid balance on postoperative day one. Vascular reactivity was assessed in a subset of the cohort. Confirmation of endothelial-specific effects of interferon-γ and interleukin-17A was performed in microvascular endothelial cells, assessing cytokine levels by enzyme-linked immunosorbent assays or trans-endothelial electrical resistance.

**Results:**

A total of 26 children were included in the analysis. Interferon-γ was inversely associated with vasoactive infusion score (*p* < 0.05), whereas interleukin-17A and interleukin-23 were associated with greater cumulative postoperative fluid balance (*p* < 0.01 and 0.03, respectively). Peak vascular reactivity is strongly associated with interferon-γ (*p* = 0.001), but not with circulating interleukin-17A. Human microvascular endothelial cell exposure to interferon-γ increased endothelial permeability and cytokine production.

**Conclusions:**

Interferon-γ and interleukin-17A may be associated with cardiovascular dysfunction in children after exposure to cardiopulmonary bypass, albeit with differential clinical features. Interferon-γ may directly impact vascular measures, while the impact of interleukin-17A may relate to fluid accumulation.

## Introduction

Cardiopulmonary bypass is required for the surgical repair and palliation of many complex congenital heart lesions. Cardiopulmonary bypass temporarily allows blood to be pumped, oxygenated, and cleared of carbon dioxide via an extracorporeal circuit to allow the surgical team to operate in a bloodless field.^1^ While cardiopulmonary bypass provides cardiopulmonary support for the patient during surgery, its use comes with postoperative risks. Exposure to cardiopulmonary bypass has been associated with several poor outcomes in children undergoing cardiac surgery, including acute kidney injury^2^ and percent fluid overload,^3–5^ postoperative hypoxaemia with prolonged need for mechanical ventilation^6^ and death.^7^ The post-cardiopulmonary bypass period often requires vasoactive support using adrenergic agonists and phosphodiesterase inhibitors, measured as a vasoactive infusion score, with escalating doses associated with poor outcomes.^8,9^ Early positive fluid balance and higher vasoactive infusion score within the first 24 hours of admission have been previously associated with poor outcomes.^5,9,10^

Mechanistically, cardiopulmonary bypass provokes a systemic inflammatory response secondary to complement activation, release of proinflammatory cytokines, and ischaemia-reperfusion injury.^11^ This is thought to cause cellular activation of the immune system, leading to endothelial cell dysfunction,^12^ capillary leak,^13^ and, in some cases, multiorgan dysfunction.^14,15^ While innovations in cardiopulmonary bypass practices such as more biocompatible surfaces, miniaturised circuits, and use of immunomodulatory therapies (i.e. perioperative steroids, cytokine adsorption, etc.) have become routine at many institutions, both the systemic inflammatory response and compensatory anti-inflammatory response that occur after cardiopulmonary bypass exposure continue to contribute to postoperative morbidity and mortality.^16,17^

Cardiopulmonary bypass exposure is associated with impacts on the adaptive immune system, such as T cells, driving alterations, and, likewise, dysfunction.^18,19^ T-cell reduction and transient population shifts of T-cell subsets after exposure to cardiopulmonary bypass have been previously described.^19,20^ The impact cardiopulmonary bypass has on T-cell activity and related cytokine profiles in children, however, remains poorly understood. Interestingly, several cytokines associated with T-cell pathways have been associated with cardiovascular dysfunction. Interleukin 17A, a cytokine produced by Th17 cells, has been associated with cardiovascular disease and endothelial dysfunction.^21,22^ Interferon-γ, a cytokine produced by Th1 and natural killer cells, is a promoter of endothelin-1 which attenuates nitric oxide release leading to greater vasoconstriction.^23^ Alterations to these cytokine profiles in response to cardiopulmonary bypass have potential to cause vascular dysfunction following cardiac surgery. Exploring these profiles and their relation to markers of vascular dysfunction in children undergoing cardiac surgery with cardiopulmonary bypass may identify novel therapeutic targets and should be further explored.

Herein, we aimed to describe cytokine profiles associated with Th1 and Th17 cell subtypes and their relationship to clinical markers of postoperative cardiovascular dysfunction. We hypothesised that interferon-γ would be associated with vasoactive infusion score, whereas interleukin-17A would be associated with greater postoperative fluid balance on postoperative day one.

## Materials and methods

### Patient recruitment

Patients, 18 years of age or less, undergoing corrective surgery for CHD requiring cardiopulmonary bypass were approached and recruited from two different quaternary children’s hospitals’ cardiovascular ICUs (Monroe Carell Jr. Children’s Hospital at Vanderbilt, November 2014 through February 2016 and Riley Children’s Hospital, October 2022 through June 2023). Written informed consent was obtained from the legal guardian(s) and assent from the patient, if applicable, prior to enrolment according to approved Institutional Review Board protocols.

An invasive blood-drawing access line was obtained as part of standard care for all participants and used to draw patient samples. Exclusion criteria included preoperative acute kidney injury as per the 2012 Kidney Disease Improving Global Outcomes definitions,^24^ or any preoperative initiation of extracorporeal support such as extracorporeal membrane oxygenation or continuous kidney replacement therapy.

### Cardiopulmonary bypass practices

Cardiopulmonary bypass practices had some differences between the two centres. Monroe Carell Jr. Children’s Hospital at Vanderbilt used a combination of conventional ultrafiltration, modified ultrafiltration, and zero-balance ultrafiltration for most cases. Riley Children’s Hospital used continuous ultrafiltration and modified ultrafiltration with rare use of zero-balance ultrafiltration. Transfusion practices on bypass were similar among the two centres. Red blood cell transfusions were provided to patients under 10 kg and when dilutional haematocrit reached a threshold of 30. Fresh frozen plasma was provided in the pump prime to patients under 10 kg and as needed. Steroids were reported if they were provided preoperatively. These were provided solely based on surgeon preference. Patients who received methylprednisolone were provided a one-time dose of 30 milligrams per kilogram of body weight on the day of surgery prior to the procedure.

### Sample processing and analysis

Preoperative whole blood samples were obtained within seven days prior to surgery and postoperative samples within 4 hours of cardiac ICU admission. Additional samples were collected at 24 hours if available. Samples were collected in potassium salt of ethylene diamine tetra-acetic acid tubes and kept on ice until processing, which occurred within 60 minutes of sample collection. Whole blood was centrifuged at 2000 × g for 15 minutes, at 4°C. Aliquoted plasma was stored in a freezer at −80°C. Samples were thawed, centrifuged, and analysed as per R&D data sheets General Enzyme-Linked Immunosorbent Assay Protocol for this study.

### Enzyme-linked immunosorbent assays

Enzyme-linked immunosorbent assays (R&D systems, Duosets) were performed according to the manufacturers recommendation for the following cytokines performed on plasma or cell culture supernatant: interleukin-12, interferon-γ, interleukin-17A, interleukin-23, interleukin-6, transforming growth factor β1, interferon protein-10, soluble vascular cell adhesion molecule, and tissue inhibitor of metalloproteinases-1 for cell supernatants. Dilution factors for enzyme-linked immunosorbent assays were set at 1:5, based on dilution testing of four patient samples (pre- and post-cardiopulmonary bypass).

### Vascular reactivity measurements

A subset of the cohort underwent laser Doppler perfusion monitoring with iontophoresis at the time of blood collection. Laser Doppler perfusion monitoring measurements were performed using a Periflux 5010 coupled to a Perilont 382b (Perimed, Stockholm, Sweden) as previously described.^25^ Briefly, 180 μL of 2% acetylcholine (Sigma-Aldrich, St. Louis, MO) was pulsed with a 0.1 mA anodal current for 20 seconds for a total of five doses separated by 120 seconds over 10 minutes. After a 10-minute rest and at a separate site, 180 μL of 1% sodium nitroprusside (Sigma) was pulsed with a 0.2 mA cathodal current using identical dosing intervals and duration.

### Clinical measurements of cardiovascular dysfunction

Cumulative postoperative fluid balance measurements over the first 24 hours were included in this study. Fluid balance was calculated using the formula (Σ Fluid In (Litres) − Fluid Out (Litres)). Percent cumulative fluid balance was calculated using this formula and dividing by the preoperative weights of all patients, and multiplying by 100 (*Σ* Fluid In (Litres) − Fluid Out (Litres)) / (preoperative weight (kg)) x100% as previously described.^26^ A weightbased formula was not utilised due to the lack of daily weight data in our cohort. Vasoactive infusion score was calculated at 24 hours postoperatively. Vasoactive infusion score was calculated using the formula (*Σ* Dopamine dose (μg/kg/minute) + Dobutamine dose (μg/kg/minute) + Milrinone dose × 10 (μg/kg/minute) þ Epinephrine dose × 100 (μg/kg/minute) + Norepinephrine dose × 100 (μg/kg/minute) + Vasopressin dose × 10,000 (U/kg/minute)).^9^ The clinical outcomes of this study were the percent fluid balance and vasoactive infusion score measured on post-operative day one after surgery.

### Cell and culture conditions

Pooled neonatal human dermal microvascular endothelial cells were purchased from Lonza (Basel, Switzerland). Human microvascular endothelial cells were grown in Vascular Cell Basal Medium (ATCC; PCS-100–030) supplemented with 5% fetal bovine serum. Cells were plated at approximately 30,000 cells/cm^2^ and grown to confluence. Experiments were conducted between the 3rd and 6th passages. Media was exchanged at least every three days.

### Trans-endothelial electrical resistance and supernatant cytokines

Trans-endothelial electrical resistance was performed using CellZScope2 (nanoAnalytics GmbH, Münster Germany). Human microvascular endothelial cells were plated on ThinWell cell culture inserts (0.4 μm pore diameter, Greiner BioOne, Monroe, NC) at a density of 40,000 cells/well and allowed to grow to confluence in medium for 24 hours. Cytokine exposure concentrations were chosen based on prior literature, and proportions were kept to reflect those of the levels found in the plasma during this study.^27,28^ A concentration of 20 ng/mL of interferon-γ, 2 ng/mL of interleukin-17A, or an equal volume of sterile water (vehicle control) was added to wells and completed in sextuplicate. Sterile water was used as a vehicle control by percent volume to ensure any effects were not a result of the reagent diluent and totalled less than one percent by volume. Hourly resistance measurements (Ω⋅cm^2^) were then collected for an additional 24 hours in the trans-endothelial electrical resistance device, which was kept incubated at 37°C and normalised to the time of agonist exposure. For cytokine measurements, cells were plated at 40,000 cells/well and allowed to reach confluency. Cells were then exposed to the same concentrations of interferon-γ, interleukin-17A or vehicle for 24 hours, after which supernatants were collected and stored at −80°C for future enzyme-linked immunosorbent assay analysis.

### Statistical analysis

Patient characteristics, including demographic data, surgical procedures, and cardiopulmonary bypass times, were collected. The change in plasma concentration (delta concentration) was defined as the difference between the post- and preoperative samples. The primary analysis was to evaluate the association between the delta cytokine concentration and clinical markers of vascular dysfunction, including vasoactive infusion score and percent fluid balance on postoperative day one after surgery. Multiple linear regression analysis was used to evaluate the delta concentration of cytokines and their association with percent fluid balance and vasoactive infusion score at this timepoint. Simple linear regression was used to compare cytokine concentrations with the peak laser Doppler perfusion monitoring response for vascular reactivity analysis. An ordinary one-way analysis of variance was used to compare cytokine activity and trans-endothelial electrical resistance results across human microvascular endothelial cell exposures to interleukin-17A, interferon-γ, and controls. All data were normalised to baseline resistance per condition prior to treatment addition for analysis. As a secondary analysis, preoperative steroid administration was compared to delta cytokine plasma concentration using unpaired t-tests. All statistics were performed using GraphPad Prism Statistical Software Version 9.0.0, and statistical significance was defined as a *p*-value < 0.05.

## Results

Twenty-six children undergoing corrective surgery for CHD requiring cardiopulmonary bypass were enrolled in the study between the two sites. The median age of participants was 150 (IQR 28–234) days. Median preoperative weight was 5.5 (IQR 4.3–6.36) kg. Patient characteristics and demographic data are summarised in Table 1. Surgical characteristics and cardiopulmonary bypass times by mortality category are included in Supplementary Table S1.

### Lymphocyte-related cytokine profiles and clinical data

Interferon-γ demonstrated a significant negative linear correlation with vasoactive infusion score (*p* = 0.038, Table 2), whereas Th17-associated cytokines interleukin-23 and interleukin-17A had a positive correlation with percent fluid balance on postoperative day one (*p* = 0.009 and <0.001, respectively, Table 3). Interleukin-12 and transforming growth factor β1 were also included given their roles in promoting differentiation of Th1 and Th17 subtypes, respectively. These were not associated with vasoactive infusion score or percent fluid balance. Notably, one patient in our cohort had significantly higher delta concentrations of interleukin-17A (149 pg/mL) and percent fluid balance on postoperative day one (54.9%). When this patient was excluded from analysis, interferon-γ remained associated with vasoactive infusion score on postoperative day one (*p* = 0.04); however, interleukin-17A and interleukin-23 lost significance with percent fluid balance on postoperative day one (*p* = 0.15).

Nine of the 26 participants received preoperative steroid administration with methylprednisolone. Delta plasma concentrations of interferon-γ were overall lower in the steroid group but did not reach statistical significance (*p* = 0.07) (Supplementary Figure S2A). However, delta plasma concentrations of interleukin17A were significantly higher in the steroid group (*p* = 0.03) (Supplementary Figure S2B). Spaghetti plot comparing individual pre- and postoperative concentrations of interferon-γ and interleukin-17A by steroid group revealed interleukin-17A concentrations were significantly higher postoperatively in those who received steroids (*p* = 0.01) (Supplementary Figure S3A-D). Total cardiopulmonary bypass time was not associated with delta plasma cytokine concentrations of interleukin-17A (*p* = 0.99) or interferon-γ (*p* = 0.90). Of the 26 participants, 11 had an additional blood sample drawn at 24 hours postoperatively. Delta plasma concentrations of interleukin-12, interferon-γ, interleukin-17A, interleukin-23, and transforming growth factor β1 at this time point were not associated with 24-hour vasoactive infusion score or percent fluid balance within this subset of our cohort.

### T-cell cytokine profiles and vascular reactivity

**Figure 1.**
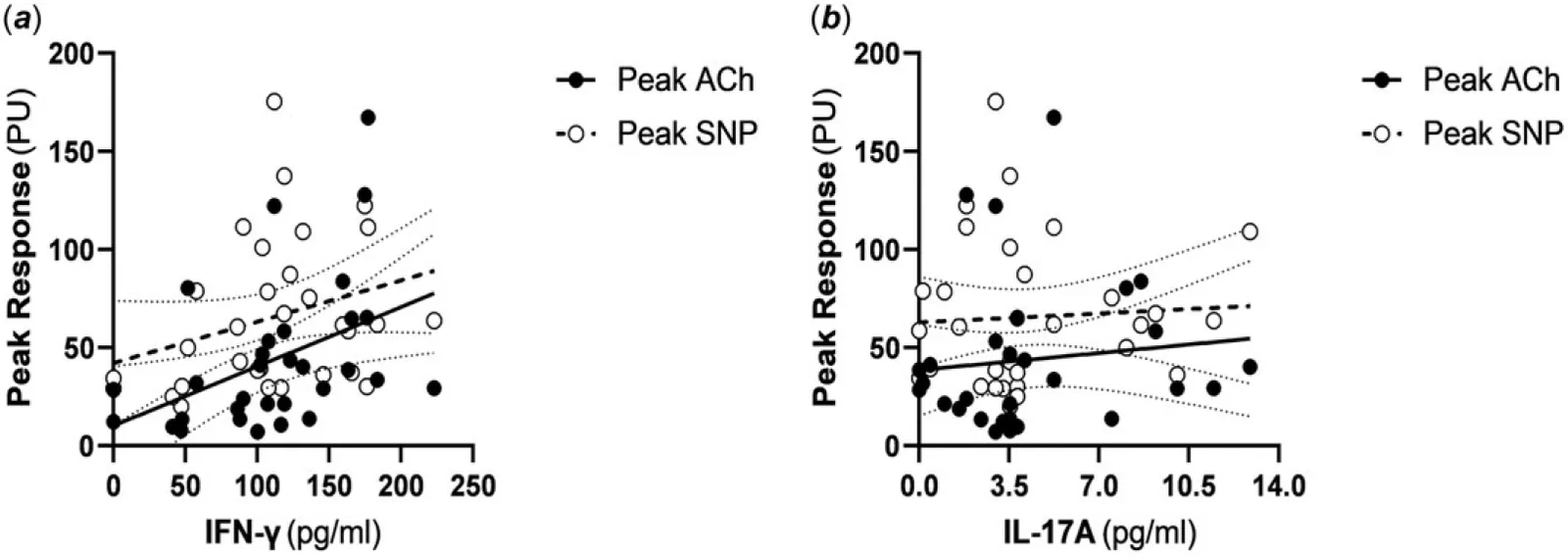
Vascular reactivity as measured by laser doppler perfusion monitoring compared to (***a***) Interferon-γ (IFN-γ) and peak acetylcholine (Ach), rs 0.55 (95% CI 0.23–0.76); sodium nitroprusside (SNP), rs 0.39 (95% CI 0.03–0.66) and (***b***) Interleukin-17A (IL-17A) (Ach, rs 0.22 (95% CI −0.15–0.54); SNP, rs 0.06 (95% CI −0.31–0.41). Linear regression with 95% confidence intervals are shown.

Given the association of interferon-γ with vasoactive infusion score, we assessed the correlation between T-cell cytokines and vascular reactivity. Vascular reactivity measurements were performed in 11 of the 26 participants. Interferon-γ correlated with peak vascular reactivity response to acetylcholine (*p* = 0.001) and sodium nitroprusside (*p* = 0.03, Figure 1A). Interleukin-17A did not correlate with peak response to acetylcholine (*p* = 0.21) or sodium nitroprusside (*p* = 0.73, Figure 1B).

### HMVEC permeability and endothelial cytokine production

**Figure 2.**
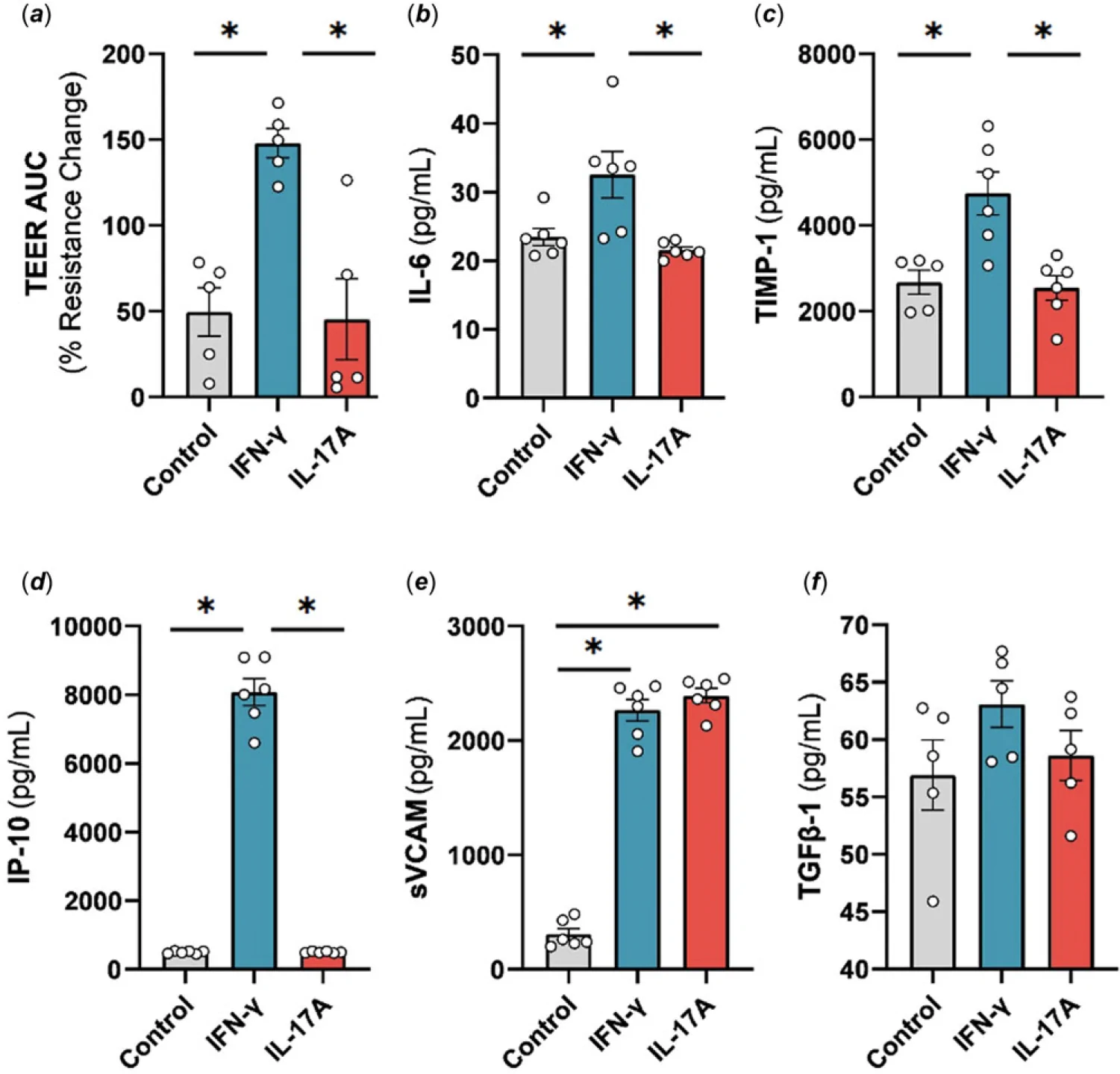
Ordinary one-way analysis of variance (ANOVA) comparing endothelial permeability (***a***) and cell supernatant cytokine concentrations (***b–f***) of human neonatal dermal microvascular endothelial cells exposed to interferon-γ (IFN-γ) (20 ng/ml) and interleukin-17A (IL-17A) (2 ng/ml) for 24 hours. IL-6 = interleukin-6; IP-10 = interferon-γ induced protein 10; sVCAM = soluble vascular cell adhesion molecule; TGF-β1 = transforming growth factor-β1; TIMP-1 = tissue inhibitor of metalloproteinase-1. *p*-values were determined by ANOVA with Tukey correction. * Denotes statistical significance as defined by *p* < 0.05.

As interferon-γ appeared to correspond to vascular function more so than interleukin-17A, we determined if interferon-γ versus interleukin-17A exposure could directly impact endothelial responses. Change in trans-endothelial electrical resistance was significantly higher in cells exposed to interferon-γ (p< 0.01) as opposed to interleukin-17A (*p* = 0.98, Figure 2A). Interferon-γ exposure to human microvascular endothelial cells caused significantly higher release of interleukin-6 (*p* = 0.02), tissue inhibitor of metalloproteinases-1 (*p* = < 0.01), and interferon protein-10 (*p* = < 0.01, Figure 2B–D) in cell supernatants. Soluble vascular cell adhesion molecule was significantly elevated in both interferon-γ and interleukin-17A exposure groups (p< 0.01, Figure 2E). There was no significant difference in transforming growth factor β1 release in either exposure group when compared to controls (Figure 2F).

## Discussion

The inflammatory state of cardiopulmonary bypass has long been accepted as a natural consequence of extracorporeal therapy that is required for the repair of many complex congenital heart lesions. Inflammation can arise from many sources and cell types, but a key aspect is the involvement of white blood cells, particularly lymphocytes. The importance of this is highlighted by the presence of T-cell responses post-operatively that have been linked to several poor outcomes, including mortality.^29^ In this multi-centre observational study exploring T-cell-related cytokines and cardiovascular dysfunction in children undergoing cardiac surgery with cardiopulmonary bypass, we uncovered that interferon-γ concentrations may be inversely associated with vasoactive infusion score, whereas interleukin 17-A may be more closely associated with fluid accumulation at 24 hours postoperatively. Further, interferon-γ exposure to human microvascular endothelial cells in vitro demonstrated a greater inflammatory response and change in endothelial barrier function as measured by trans-endothelial electrical resistance.

Differential responses to cytokine profiles were a key finding of this study. Interferon-γ release after bypass exposure showed a more direct impact on patients’ vascular function in the critical postoperative period. This was supported by the association of Interferon-γ with lower need for vasoactive support, greater endothelial permeability, and strong peak vascular reactivity response in our study. Previous studies exploring the impacts of Interferon-γ on vascular function have also shown complex relationships.^30,31^ Regulatory roles such as endothelial barrier function, Th1/Th2 balance, and cellular proliferation may have differential impacts on the vasculature.^32^ While the cytokines measured, such as interferon-γ, are not specific to T cells and may be produced by other cell types, they have most often been linked to T-cell differentiation and activation.^33^ Other potential sources of interferon-γ include natural killer cells, macrophages, and neutrophils.^34–36^ In this regard, we also tested Th17-associated cytokine profiles to assess whether a shared clinical phenotype exists.

Interleukin-17A has been previously associated with vascular inflammation and the development of acute kidney injury.^22,37,38^ Mechanistically, interleukin-17A may lead to kidney inflammation, injury, and oliguria, which could explain why interleukin-17A had a greater relationship with fluid accumulation on postoperative day one without demonstrating increased endothelial permeability on trans-endothelial electrical resistance analysis. Interestingly, plasma transforming growth factor β1 concentration was not associated with vasoactive infusion score or percent fluid balance. As a cytokine involved in the early Th17 differentiation pathway, transforming growth factor β1 has also been shown to inhibit interferon-gamma secretion,^39^ which could play a role in regulating the two potential phenotypes described in this study. Given this cohort was too small to accurately measure secondary clinical outcomes such as acute kidney injury or major adverse kidney events, future large studies should explore interleukin-17A and its relationship to kidney-specific outcomes in children receiving cardiac surgery with cardiopulmonary bypass.

Excessive immune activation induced by cardiopulmonary bypass has been a longstanding concern in the post-operative management and outcomes of CHD. Indeed, our data demonstrate that bypass-associated inflammation may potentiate distinct postoperative phenotypes. Interferon-γ may predominantly affect the vasculature and interleukin-17A may have a more indirect impact on the vasculature through impacting postoperative fluid balance. While several strategies have been proposed to minimise systemic inflammation due to cardiopulmonary bypass,^40^ the approach has always been broad and untargeted. A recent large randomised controlled trial exploring methylprednisolone administration preoperatively to mitigate cardiopulmonary bypass-associated inflammation revealed steroid exposure did not significantly reduce the likelihood of a worse outcome.^41^ Further, generalised cytokine adsorption has failed to show positive effects on outcomes such as mortality after cardiopulmonary bypass surgery.^42^ These findings suggest that generalised immune-suppression may not be beneficial. From a cytokine perspective, steroid administration in our study was associated with interleukin-17A elevations and decreases in interferon-γ concentrations nearing significance. As a next step, future efforts may consider exploring these cytokines as they relate to postoperative outcomes including acute kidney injury and operative mortality in larger studies. Additionally, the implications of this study may also be potentially applicable to a broader scope of critically ill patients, as similar cytokine triggers can be seen with the use of extracorporeal membrane oxygenation in the ICU.^43^

Interferon-γ has previously been shown to be reduced after cardiac surgery with cardiopulmonary bypass.^44,45^ In our study, we found variable changes to interferon- γ concentration and differential impacts of interferon-γ on the vasculature. Interferon-γ after bypass demonstrated an inverse relationship to vasoactive infusion score on postoperative day one (greater suppression of interferon-γ was associated with increased vasoactive requirement). This could suggest interferon-γ suppression postoperatively has detrimental effects on the macrovasculature. In contrast, our in vitro experiments exploring its effects on human microvascular cells revealed a greater inflammatory response with higher interleukin-6 production and greater loss of endothelial barrier function on trans-endothelial electrical resistance. This could suggest elevated interferon-γ may cause harmful effects on the microvasculature. Given this data, we propose that too much or too little interferon-γ postoperatively may have negative effects, and a balance between these two responses may be optimal. The use of interferon-γ targeted therapies focused on inhibition, such as Emapalumab or janus kinase inhibitors, or supplementation, such as human recombinant interferon-γ, may provide benefit based on each patient’s individual immune response to bypass. This approach may also avoid increasing interleukin-17 which was associated with steroids in our and previous studies.^46^ These data may be used to guide future trials focused on personalised medicine approaches to bypass-mediated inflammation.

Interestingly, recent data have shown that bypass-associated injury to the endothelial glycocalyx is associated with greater fluid accumulation postoperatively in children undergoing high-risk cardiac surgeries.^47^ Matrix metalloproteinases have been shown to cause glycocalyx degradation, and tissue inhibitor of metalloproteinases-1 is a natural inhibitor of metalloproteinases. This could provide an alternative explanation for why endothelial cell production of tissue inhibitor of metalloproteinases-1 was lower in cells exposed to interleukin-17A than to interferon-γ, and a higher percent fluid accumulation on postoperative day one was more closely linked to greater delta interleukin-17A concentration; however, more conclusive data is needed.

There were several limitations to this study. Our sample size was small, limiting our ability to assess postoperative outcomes in our cohort because of their low incidence. We also included a relatively heterogeneous paediatric population with regard to age and surgical procedure performed. Additionally, while the cytokines measured in this study are involved in Th1 and Th17 T-cell subtypes, they are not specific to these processes and play other roles in regulating the immune system. As such, we cannot definitively say that these cytokine profiles result solely from changes in T-cell subtype differentiation, as other immune cells may also contribute. Further, trans-endothelial electrical resistance was used as a surrogate of endothelial permeability in this study. While this has been shown to be a strong indicator of endothelial barrier function in vitro, the physiologic endothelial response to T-cell cytokines across different vascular beds in patients may differ. Lastly, while this study explored T-cell-associated cytokine profiles and their effects on vascular dysfunction, a more detailed characterisation of lymphocyte populations to vascular outcomes is necessary after cardiopulmonary bypass.

In conclusion, our multicentre translational study exploring T-cell-related cytokines in children undergoing cardiac surgery with cardiopulmonary bypass revealed that interferon-γ and interleukin-17A may have an impact on postoperative cardiovascular dysfunction. Interferon-γ may have a more direct effect on vascular function, whereas interleukin-17A may indirectly affect vascular function through postoperative fluid accumulation. Future investigation in a larger sample of patients is needed to better delineate the role these cytokines have on postoperative outcomes in children undergoing cardiac surgery with cardiopulmonary bypass.

## Supplementary Material

1

2

3

**Supplementary material.** The supplementary material for this article can be found at https://doi.org/10.1017/S1047951126111779.

## Figures and Tables

**Table 1.** Patient characteristics

Characteristic	*N* = 26 (IQR, %)

Age (days)	150 (28, 234)
0–30 days	8
31–365 days	15
1–3 years	1
>3 years	2
Gestational age	
Preterm (<37 weeks gestation)	4 (15.3)
Sex	
Male	11 (42.3)
Preoperative weight (kg)	5.5 (4.3, 6.36)
Cumulative percent fluid balance	
Postoperative day 1	413 (242, 806)
Vasoactive infusion score	
Postoperative day 1	5 (3, 8)
STAT category	
STAT1	3 (11.5)
STAT2	4 (15.4)
STAT3	9 (34.5)
STAT4	5 (20.8)
STAT5	5 (20.8)
CPB duration in minutes	142 (99, 186)
Preoperative steroids	
Yes	8 (30.8)
Single ventricle morphology	
Yes	9 (34.6)

**Table 2.** Multiple linear regression analysis of plasma t-cell cytokines and vasoactive infusion score on postoperative day one

T-cell cytokines	Median (IQR)	Coefficient	Std. error	*t*-ratio	*p* Value

Th1 cytokines					
Delta IL-12	3.1 (0.6–7.4)	−0.05	0.108	−0.46	0.65
Delta IFN-*γ*	10.2 (−53.9–46.8)	−0.012	0.005	−2.199	0.038
Th17 cytokines					
Delta TGF-*β*1	5.2 (0.6–8.2)	−0.018	0.066	−0.27	0.79
Delta IL-23	1.5 (−26.5–44.2)	0.0002	0.0004	0.52	0.61
Delta IL-17A	4.0 (−0.3–9.9)	0.007	0.035	0.19	0.85

**Table 3.** Multiple linear regression analysis of plasma t-cell cytokines and cumulative percent fluid balance on postoperative day one

T-cell cytokines	Median (IQR)	Coefficient	Std. error	*t*-ratio	*p* Value

Th1 cytokines					
Delta IL-12	3.1 (0.6–7.4)	−0.49	0.389	1.25	0.22
Delta IFN-*γ*	10.2 (−53.9–46.8)	−0.01	0.022	0.49	0.63
Th17 cytokines					
Delta TGF-*β*1	5.2 (0.6–8.2)	0.22	0.242	0.93	0.36
Delta IL-23	1.5 (−26.5–44.2)	0.004	0.001	2.837	0.009
Delta IL-17A	4.0 (−0.3–9.9)	0.318	0.049	6.452	<0.001
