# Supplementary figures and images for: Interferon-γ and interleukin-17A associations with vascular dysfunction following paediatric cardiac surgery with cardiopulmonary bypass
Source: Cardiol Young. Author manuscript; Available in PMC 2026 Jun 3. (PMC13231944; doi:10.1017/S1047951126111779)

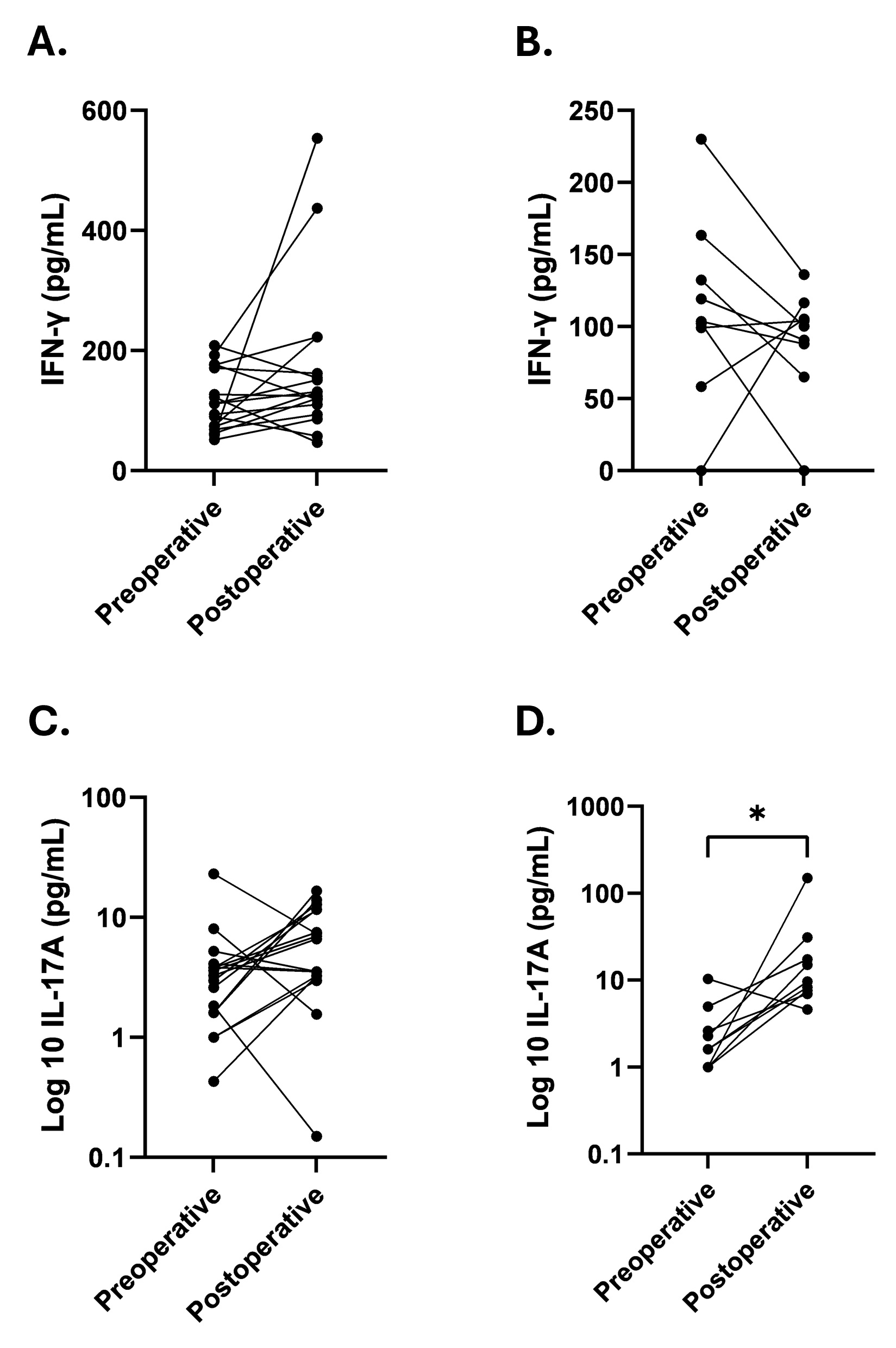

Supplement: 1 [file NIHMS2152953-supplement-1.tiff]

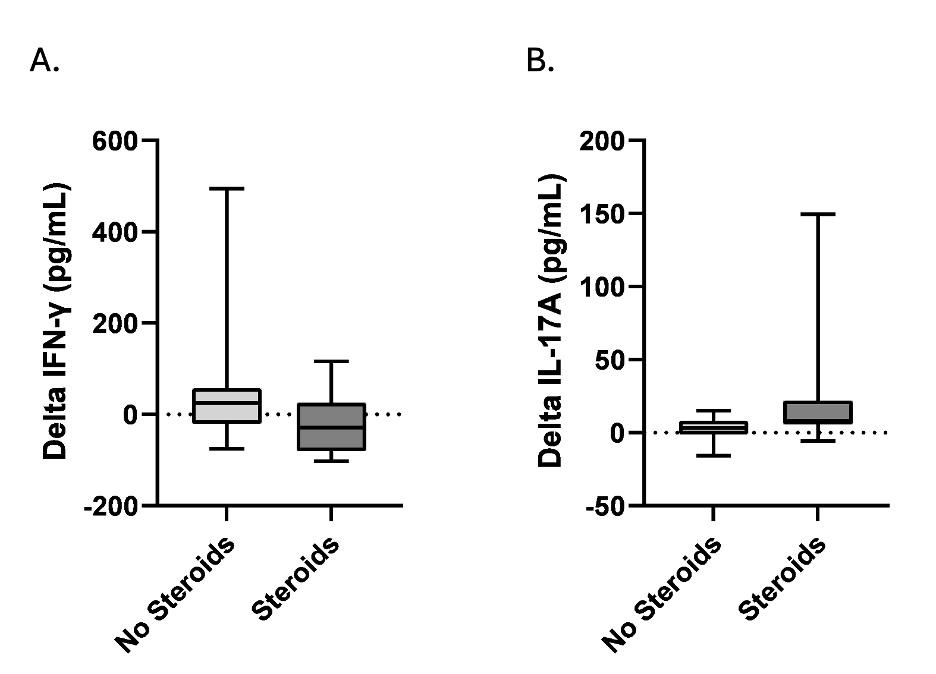

Supplement: 3 [file NIHMS2152953-supplement-3.tiff]
