# Supplementary material for: Interferon-γ and interleukin-17A associations with vascular dysfunction following paediatric cardiac surgery with cardiopulmonary bypass
Source: Cardiol Young. Author manuscript; Available in PMC 2026 Jun 3. (PMC13231944; doi:10.1017/S1047951126111779)
Supplement: 2 [file NIHMS2152953-supplement-2.docx]

**Supplementary Table S1.** **Surgical Procedures and Cardiopulmonary Bypass Duration by STAT Category**

| Procedure/ Category | *N* = 26 |
| --- | --- |
| **STAT 1**  VSD repair  ASD repair  **STAT 2**  Glenn repair  Complete AVC repair | **3**  2  1  **4**  1  3 |
| **STAT 3**  TOF repair with RV to PA conduit  DORV with intraventricular tunnel  Arterial switch with VSD closure  Mitral valvuloplasty to valve replacement  **STAT 4**  Bilateral unifocalization MAPCA’s  Fontan revision  TAPVC repair  Yasui procedure  **STAT 5**  Norwood procedure  Unifocalization MAPCA’s and central shunt | **9**  3  2  3  1  **5**  2  1  1  1  **5**  4  1 |
| **CPB duration in minutes**  STAT category 1 (n= 3)  STAT category 2 (n= 4)  STAT category 3 (n= 9)  STAT category 4 (n= 5)  STAT category 5 (n= 5) | 142 (99, 186)  107 (80, 137)  164 (122, 189)  118 (78, 181)  174 (99, 208)  158 (139, 184) |
